# Diabetes quality of life perception in a multiethnic population

**DOI:** 10.1007/s11136-014-0885-3

**Published:** 2014-12-10

**Authors:** S. G. K. Goh, B. N. Rusli, B. A. K. Khalid

**Affiliations:** Jeffrey Cheah School of Medicine and Health Sciences, Monash University Malaysia, Jalan Lagoon Selatan, 46150 Bandar Sunway, Selangor Darul Ehsan Malaysia

**Keywords:** Quality of life, Diabetes, Perception, Sexual dysfunction, Asians

## Abstract

**Aim:**

The aim of this study was to determine ethnic differences and predictors of the perception of quality of life (QOL) in a multiethnic Malaysian population with type 2 diabetes.

**Methods:**

A population-based cross-sectional study was done in three different states in Malaysia. The Asian Diabetes Quality of Life (AsianDQOL) tool specific for type 2 diabetes is the primary outcome tool. One-way analysis of covariance was undertaken to examine ethnic differences on the total and component AsianDQOL scores controlling for important covariates. Stepwise multiple linear regression models were used for selecting predictors for the AsianDQOL score with stratification for ethnicity and language.

**Results:**

A total of 647 subjects (338 Malays, 160 Chinese and 149 Indians) were recruited. Chinese scored significantly lower (78.1 ± 11.6) on the AsianDQOL (total) score compared to Malays (81.4 ± 9.0) and Indians (81.5 ± 9.2) (*F* = 3.060, *p* = 0.049, *η* ^2^ = 0.02). Likewise, Chinese scored significantly lower (21.0 ± 4.3) on the AsianDQOL (diet) score compared to Malays (22.8 ± 3.6) and Indians (22.5 ± 3.7) (*F* = 4.96, *p* = 0.008, *η* ^2^ = 0.04). The main predictors of AsianDQOL (total) score for the English language group of different ethnicities were sexual dysfunction (−4.5), having visual problems (−3.7), female (−2.8) and glycemic control (−1.6). Sexual dysfunction was negatively correlated with QOL in Malay, Chinese ethnic group and Indian ethnic groups.

**Conclusion:**

The perception of AsianDQOL is different across ethnic groups and languages spoken. Significant differences in the English-speaking group and the non-English-speaking group are detected within the same ethnicity. Sexual dysfunction severely impacts AsianDQOL in a multiethnic Asian population and remains an important determinant regardless of ethnicity and language.

## Introduction

Quality of life (QOL) measurement apart from physical indices or glycemic control is becoming increasingly important with rapid progression in the field of medicine. QOL has become a crucial outcome measure for management of diabetes (DM). The current available QOL tools are divided into generic and disease specific. In Asia, most of the QOL tools were translated from those developed based on the Western population [1, 2]. The translated versions were then validated for use in the local Asian population [3, 4]. A review of the translation and adaptation process of QOL tools in Asian countries show only 24 % measured the local conception of QOL [5].

QOL is a broad concept, and perception of QOL can be affected by different factors such as the socioeconomic status, culture, population group and even ethnicity [4, 6, 7]. Lau et al. [6] 1998 studied the self-perceived QOL of Chinese elderly people in Hong Kong and concluded that general commonalties of health, life satisfaction and social relationships with studies done in Western countries. However, differences in the characteristics and ranking of components are present and must be adjusted or modified for a better reflection of QOL. Several population-based studies in Singapore concluded that ethnicity and socioeconomic status are important in determining QOL in a multiethnic Asian population [4, 7]. There are limited data on the impact of westernization on the perception of QOL in a multiethnic population. The preferred language of the subject reflecting his or her upbringing may determine the impact of westernization on South East Asian countries Malaysia, Singapore, Brunei, Indonesia and the Philippines. Those who preferred English language tend to be English educated locally or overseas and have a higher exposure to Western culture and lifestyle compared to the more traditional group who are still following local customs, lifestyles and beliefs.

Malaysia’s population is similar to Singapore in terms of the different ethnic group composition. The education system in Malaysia practices bilingual concept, resulting in a majority of Malaysians who are proficient in more than one language. Their preferred language is mainly influenced by the medium of education and influence of family and social network [8, 9]. Whorf in 2012 explained that language guides our cognition and shaped our conceptual knowledge, and subsequently, there is strong evidence, supporting the theory that language directs thoughts and behavior in human beings [10, 11]. We hypothesized that a population of the same ethnicity but with different preferred language will not share the same perception of QOL. We also want to determine the factors influencing QOL and the impact of DM on QOL in our population with diverse ethnicities and languages.

## Methods/design

### Study design

A population-based cross-sectional study was conducted across the three most populous states in Peninsular Malaysia. The minimum sample size was calculated for multiple regression based on recommendations by Knofczynski et al. [12] in 2007. The recommended minimum sample size of good prediction level with five predictor variables and medium population correlation coefficients of 0.50 were 65 subjects [12]. The Malaysian statistics department states that of the 28 million population, 60 % are Malays, 23 % Chinese, 7 % Indians and 10 % others. The prevalence of diabetes in Malaysia in 2013 was highest in Indian ethnic group (38 %) compared to Malays (24 %) and Chinese (18 %) [13]. Recruitment sampling takes into consideration these two factors. Convenient sampling method was used, but in order to reduce bias and to cater for the above variables, subjects were recruited from different levels of healthcare facilities across three states in Malaysia. The healthcare system in Malaysia is divided into the private sector and the government section. The government sector is free for all Malaysians while patients in the private sectors will have to bear their own expenses if they are not covered by insurance. According to the Malaysia Health System review by WHO Western Pacific Region in 2012, the government sector (free or subsidized fee) covers 82 % of inpatient and 35 % of outpatient care, while the private sector (self-paying or third party paying) covers 18 % of inpatient and 62 % of outpatient care [14]. The study sample gives a fair representative with 56 % from government sector, 36 % from private sector and 4 % of both. Subjects were also recruited from clinic and hospital with consultants, internal medicine specialist and general practitioner clinics. This is to ensure comprehensive coverage of subjects from all different socioeconomic background and levels of medical care. Subjects were of major ethnic groups residing in Malaysia (Malay, Chinese and Indian) with different preferred language, e.g., English-speaking group of Malay ethnicity. This will ensure a fair representative sample of the Malaysian population who has DM. Patients must have at least 6 years of formal education. The literacy rate in Malaysia (2008–2013) is 93 % [15]. Our study population comprised of the working group and non-working group that consisted of retirees, housewives, students and unemployed. Subjects were from three most populous states in Malaysia (Selangor, Johor and Wilayah Persekutuan), which covered the urban and suburban group. The study population also included patients with long-standing DM (more than 5 years), the newly diagnosed and those who are diagnosed less than 5 years with or without DM complications, on different types of treatment and levels of glycemic control.

The inclusion criteria are subjects with type 2 DM, with or without pharmacological treatment, above 18–80 years old, completed at least 6-year education and able to give written consent. The exclusion criteria were concurrent Parkinson’s disease, Alzheimer’s disease, dementia and severe visual impairment. The nature of the study was explained to the subjects and a copy of the information consent form. Subjects consenting to the study were given a copy of AsianDQOL in their preferred language to fill. Thorough medical history and physical examination were done by the researcher. Physical examination includes measurement of height, weight, blood pressure, waist circumference body mass index (BMI) and other complications of diabetes. Anthropometric measurements were taken according to the World Health Organization (WHO) guidelines [16]. BMI was calculated as weight (kg) divided by height (m^2^). The waist circumference was taken as between the lowest rib margin and iliac crest. Blood sample for HbA1c levels was taken via venous sampling, and analysis was done using the Arkray Adams HA-8160 (Arkray, Inc., Nakagyo-ku, Kyoto, Japan) Diabetes Control and Complications Trial aligned cation-exchange chromatographer analyzer for HbA1c. Ethnics clearance was granted by the Monash University Human Research Ethics Committee (MUHREC), approval no CF2630–2011001537. Written informed consent was obtained from all participants.

### Primary outcome tool

In our previous study, we have developed and validated a QOL assessment tool specific for type 2 DM in Asia. The tool (AsianDQOL) was constructed based on the diverse ethnicity, culture, language, religion and sociodemographic in Malaysian population. This tool shows good reliability and is available in English, Malay/Indonesian language and Chinese-Mandarin. The English and Malay language questionnaire had five components and 21 items, while the Chinese-Mandarin language version had five components with 18 items. The questionnaires showed good reliability with Cronbach’s alpha’s score of >0.7. Among the domains assessed were diet and eating habits, emotion and self-care, memory and cognition, financial aspects and interpersonal relationships. The scoring system of this questionnaire is such that each component can be assessed individually or as total score. Based on the total score, the subjects can be classified as having ‘excellent QOL,’ ‘good QOL,’ ‘moderate QOL’ or ‘poor QOL.’

### Statistical analyses

Data were analyzed using the Statistical Package For Social Sciences (SPSS) version 20. A one-way ANOVA was used to analyze mean differences across ethnic groups; Chi-square test was used to analyze categorical differences across ethnic groups. A one-way analysis of covariance (ANCOVA) was used to compare ethnic differences of the AsianDQOL (total) and component scores controlling for significant covariates. Stepwise multiple linear regressions were used to identify significant predictors of the AsianDQOL total score across ethnicities and languages. Statistical significance was set at *p* < 0.05.

## Results

The total number of subjects recruited was 704, 57 (8 %) subjects were removed due to incomplete data and the final number for analysis was 647. Table 1 depicts the demographic data, comorbidities and treatment characteristics of the study population by the different ethnic groups. Chinese scored significantly lower (78.1 ± 11.6) on the AsianDQOL (total) score compared to Malays (81.4 ± 9.0) and Indians (81.5 ± 9.2) (*F* = 3.060, *p* = 0.049, *η* ^2^ = 0.02). Likewise, Chinese scored significantly lower (21.0 ± 4.3) on the AsianDQOL (diet) score compared to Malays (22.8 ± 3.6) and Indians (22.5 ± 3.7) (*F* = 4.96, *p* = 0.008, *η* ^2^ = 0.04). A preliminary one way between groups ANCOVA was conducted to analyze the effect of ethnicity and different covariates such as age, gender, working status, duration of DM, education level, types of DM treatment, DM care center and HbA1c on AsianDQOL score. Only HbA1c was significant (Table 2). ANCOVA was used to compare the effect of ethnicity on the perception of QOL. The independent variable was the ethnic groups (Malay, Chinese and Indian), and the dependent variable was the QOL score measured by AsianDQOL. Participant’s HbA1c level was used as the only covariate in this analysis. Preliminary checks were conducted to ensure that there was no violation of the assumptions of normality, linearity, homogeneity of variances, regression slopes and reliable measurement of the covariate. Analysis showed significant differences between the ethnic groups on QOL (*F* = 3.060, *p* = 0.049, *η* ^2^ = 0.02) (Table 2). When we analyzed the different components’ score as dependent variable, only the diet component showed significant differences between the ethnic groups. This indicates that the perception on diet was different between the ethnic groups (Table 3). The other components such as relationship, memory, energy level and financial were not significant (Table 3). Stepwise multiple linear regressions on the 647 subjects to analyze the possible contributors to QOL score were done. Linear regression done on 292 Malay-language-speaking (MLS) subjects of different ethnicities (255 Malays, 32 Indians and 5 Chinese) showed longer duration of DM, and sexual dysfunction is negatively associated with QOL score (Table 4). The equation generated, QOL = 90 (constant) − 1.8 (duration of DM) − 5.9 (sexual dysfunction). For example, for a subject with diabetes for 10 years with complication of sexual dysfunction would obtain a score of 66. Excluded variables were age, gender, education level, glycemic control (HbA1c), BMI, ethnicity, comorbidities such as heart disease, hyperlipidemia, hypertension, visual problems and nerve problems (Table 4).

**Table 1:** Demographic, comorbidities and treatment characteristics of the Malay, Chinese and Indians with type 2 diabetes mellitus

Characteristics	Malay No. (%)	Chinese No. (%)	Indian No. (%)	*χ* ^2^	df	*p*
Age 0(mean ± SD) (year)	55.0 ± 11.6	57.5 ± 11.2	54.5 ± 10.9			0.24^a^
Gender				2.90	2	0.41
Men	214 (63.3)	103 (64.4)	106 (71.1)			
Women	124 (36.7)	57 (35.6)	43 (28.9)			
Language				453.80	4	0.00
Malay	255 (75.4)	5 (3.1)	32 (21.5)			
English	83 (24.6)	82 (51.2)	117 (78.5)			
Mandarin	0 (0.0)	73 (45.6)	0 (0.0)			
Marital status				4.69	2	0.20
Married	304 (89.9)	133 (83.1)	130 (87.2)			
Not married	34 (10.1)	27 (16.9)	19 (12.8)			
Education level				14.40	2	0.03
Secondary school	137 (40.7)	54 (34.2)	44 (29.5)			
Tertiary and above	200 (59.3)	104 (65.8)	105 (70.5)			
Occupation				16.10	2	0.00
Working	191 (56.5)	91 (56.9)	111 (74.5)			
Not working/retired	147 (43.5)	69 (43.1)	38 (25.5)			
Comorbidities						
Hypertension	198 (58.6)	97 (60.6)	75 (50.3)	4.00	2	0.26
Hyperlipidemia	167 (49.4)	87 (54.4)	70 (47.0)	1.90	2	0.60
Cardiac disease	70 (20.7)	28 (17.5)	37 (24.8)	2.60	2	0.46
Visual problems	96 (28.4)	47 (29.4)	44 (29.5)	4.40	2	0.22
Nerve problems	101 (29.9)	39 (24.4)	48 (32.2)	2.80	2	0.43
Sexual dysfunction	140 (41.4)	58 (36.3)	59 (39.6)	2.00	2	0.58
Peripheral vascular disease	5 (1.5)	4 (2.5)	3 (2.0)	8.50	2	0.04
Renal problems	12 (3.6)	7 (4.4)	9 (6.0)	2.40	2	0.50
Type of treatment				19.90	8	0.34
Diet therapy alone	22 (6.5)	3 (1.9)	7 (4.7)			
Oral pills only	205 (60.7)	110 (68.8)	92 (61.7)			
Insulin only	28 (8.3)	7 (4.4)	13 (8.7)			
Oral pills and insulin	53 (15.7)	26 (16.3)	24 (16.1)			
Not on any treatment	6 (1.8)	5 (3.1)	3 (2.0)			
Duration of diabetes				6.94	4	0.64
Less than 1 year	47 (13.9)	18 (11.2)	19 (12.8)			
Between 1 and 5 years	98 (29.1)	40 (25.0)	45 (30.2)			
>5 to <10 years	80 (23.8)	48 (30.0)	31 (20.8)			
More than 10 years	112 (33.2)	54 (33.8)	54 (36.2)			
Center for diabetes care				27.50	4	0.74
Government sector	195 (60.2)	79 (52.3)	86 (59.7)			
Private sector	115 (35.5)	64 (42.4)	54 (37.5)			
Government and private sector	14 (4.3)	8 (5.3)	4 (2.8)			
HbA1c levels				14.30	6	0.11
Less than 6.5 %	63 (19.4)	29 (19.8)	32 (23.0)			
Between 6.5 and 7.5 %	73 (22.5)	48 (32.9)	42 (30.2)			
Between 7.6 and 8.5 %	67 (20.7)	26 (17.8)	19 (13.7)			
More than 8.5 %	121 (37.4)	43 (29.5)	46 (33.1)			

^a^One-way ANOVA used

**Table 2:** One-way analysis of covariance with AsianDQOL (total) score as dependent variable

Independent variable	Mean ± SD	Tests of between-subject effects			Estimated marginal means		
		*F*	*p*	*η* ^2^	Mean	95 % CI	
						Lower	Upper
Ethnic group		3.06	0.049	0.02			
Malay (*n* = 80)	81.4 ± 9.0				81.50	79.34	83.65
Chinese (*n* = 77)	78.1 ± 11.6				78.17	75.98	80.37
Indian (*n* = 114)	81.5 ± 9.2				81.38	79.57	83.18
HbA1c status		8.32	0.004	0.03			

Dependent variable: AsianDQOL (total) score. Levene’s test of equality of error variance: *F* = 2.9 (*p* = 0.056) gender (*F* = 0.19, *p* = 0.667), age (*F* = 0.31, *p* = 0.577), working status (*F* = 1.80, *p* = 0.182), duration of DM (*F* = 2.15, *p* = 0.143), education level (*F* = 0.01, *p* = 0.931), types of treatment (*F* = 0.00, *p* = 0.992) and DM care center (*F* = 0.01, *p* = 0.920)

**Table 3:** One-way analysis of covariance with component score as dependent variable

Independent variable	Mean ± SD	Tests of between-subject effects			Mean	Estimated marginal means	
		*F*	*p*	*η* ^2^		95 % CI	
						Lower	Upper
Diet component							
Ethnic group		4.96	0.008	0.04			
Malay (*n* = 80)	22.8 ± 3.6				22.88	22.06	23.69
Chinese (*n* = 77)	21.0 ± 4.3				21.08	20.25	21.91
Indian (*n* = 114)	22.5 ± 3.7				22.39	21.71	23.08
HbA1c status		20.90	0.000	0.07			
Relationship component							
Ethnic group		1.97	0.141	0.02			
Malay (*n* = 80)	8.1 ± 3.2				8.07	7.33	8.81
Chinese (*n* = 77)	8.4 ± 3.7				8.41	7.66	9.16
Indian (*n* = 114)	9.0 ± 3.2				9.01	8.39	9.63
HbA1c status		1.19	0.276	0.00			
Memory component							
Ethnic group		2.37	0.096	0.02			
Malay (*n* = 80)	16.6 ± 2.1				16.59	16.08	17.10
Chinese (*n* = 77)	16.1 ± 2.4				16.13	15.61	16.65
Indian (*n* = 114)	16.9 ± 2.4				16.88	16.45	17.30
HbA1c status		0.03	0.865	0.00			
Energy-level component							
Ethnic group		2.20	0.113	0.02			
Malay (*n* = 80)	12.5 ± 2.1				12.55	11.94	13.16
Chinese (*n* = 77)	11.7 ± 3.0				11.75	11.12	12.37
Indian (*n* = 114)	11.8 ± 3.0				11.80	11.29	12.31
HbA1c status		0.56	0.456	0.02			
Financial component							
Ethnic group		0.52	0.593	0.00			
Malay (*n* = 80)	21.4 ± 3.6				21.42	20.54	22.30
Chinese (*n* = 77)	20.8 ± 4.5				20.81	19.91	21.70
Indian (*n* = 114)	21.4 ± 4.0				21.30	11.29	22.04
HbA1c status		9.69	0.002	0.04			

Levene’s test of equality of error variances: Diet component (*F* = 1.60, *p* = 0.200), relationship component (*F* = 1.60, *p* = 0.200), memory component (*F* = 1.27, *p* = 0.282), energy-level component (*F* = 3.95, *p* = 0.200), financial component (*F* = 1.01, *p* = 0.370)

**Table 4:** Predictors of AsianDQOL (total) score stratified by language: stepwise multiple linear regression

Predictor variable	*β* coefficient		95 % CI		*p*
	Unstandardized	Standardized	Lower	Upper	
Malay/Indonesian (*n* = 292, *R* ^2^ = 0.11)					
Constant	90.30		86.90	93.71	0.000
Duration of diabetes	−1.82	−0.18	−2.98	−0.66	0.002
Sexual dysfunction	−5.87	−0.26	−8.41	−3.33	0.000
English (*n* = 282, *R* ^2^ = 0.11)					
Constant	90.95		86.24	95.65	0.000
HbA1c	−1.56	−0.18	−2.59	−0.54	0.000
Gender	−2.77	−0.13	−5.35	−0.18	0.036
Visual problems	−3.65	−0.16	−6.40	−0.90	0.010
Sexual dysfunction	−4.49	−0.18	−6.95	−2.03	0.000
Mandarin (*n* = 73, *R* ^2^ = 0.22)					
Constant	71.45		65.94	76.96	0.000
Hyperlipidemia	4.56	0.27	0.47	8.65	0.029
HbA1c	−1.85	−0.26	−3.55	−0.15	0.034
Sexual dysfunction	−5.87	−0.33	−10.10	−1.65	0.007

Analysis of the 282 English language cohort entailing 83 subjects of Malay ethnicity, 82 Chinese and 117 Indian showed the following equation (Table 4): QOL = 91 (constant) −1.6 (HbA1c) − 2.8 (female) − 3.7 (visual problems) − 4.5 (sexual dysfunction). For example, a male subject with HbA1c score of 10 with sexual dysfunction will score 70.5. This result demonstrates that having poorer glycemic control, the presence of visual problems and sexual dysfunction predicts a poorer QOL score. When compared to Malay language group, the duration of DM is no longer a determinant of QOL score. Excluded variables were glycemic control (HbA1c), education level, ethnicity, BMI, duration of DM and comorbidities such as hyperlipidemia, heart disease and kidney disease.

Analysis of the 73 Mandarin language group of Chinese ethnicity showed glycemic control, and sexual dysfunction is negatively associated with QOL score, while hyperlipidemia is associated with better QOL score (Table 4). QOL = 71 (constant) + 4.6 (hyperlipidemia) − 1.9 (HbA1c) − 5.9 (sexual dysfunction). For example, a subject with HbA1c of 10 and associated hyperlipidemia and sexual dysfunction will score 50.7. Excluded variables were education level, duration of DM and comorbidities such as hypertension, heart disease, visual problems, nerve and kidney problem.

Analysis of 255 more traditional MLS of Malay ethnicity cohort generated an equation of 98 (constant) − 2.3 (duration of DM) − 6.0 (sexual dysfunction). Sexual dysfunction and duration of DM negatively predict QOL in this group. The *R* ^2^ value was 0.14, indicating that the model explained 14 % of total QOL score (Table 5). Analysis of the 83 subjects of the English speaking of Malay ethnicity group showed diabetes renal problems was associated with worse QOL score (Table 5). QOL = 82 (constant) − 22 (renal problems). The *R* ^2^ value was 0.13, indicating that the model explained 13 % of the total QOL score. For example, a man of Malay ethnicity with Malay language as his lingua franca and has diabetes for 10 years with chronic kidney disease will obtain a score of 75, while a man of Malay ethnicity with English language as his lingua franca and has diabetes for 10 years with chronic kidney disease will obtain a score of 60.

**Table 5:** Predictors of AsianDQOL (total) score stratified by ethnicity and language: stepwise multiple linear regression

Predictor variable	*β* coefficient		95 % CI		*p*
	Unstandardized	Standardized	Lower	Upper	
Malay ethnicity Malay language (*n* = 255, *R* ^2^: 0.14)					
Constant	97.98		88.44	95.53	0.000
Duration of diabetes	−2.28	−0.23	−3.49	−1.07	0.000
Sexual dysfunction	−6.01	−0.27	−8.68	−3.34	0.000
Malay ethnicity English language (*n* = 83, *R* ^2^: 0.13)					
Constant	81.79		79.81	83.76	0.000
Renal problems	−21.79	−0.37	−34.24	−9.26	0.001
Chinese ethnicity Mandarin language (*n* = 73, *R* ^2^: 0.21)					
Constant	71.45		65.94	76.96	0.000
Hyperlipidemia	4.56	0.27	0.47	8.65	0.029
HbA1c	−1.85	−0.26	−3.55	−0.15	0.034
Sexual dysfunction	−5.87	−0.33	−10.10	−1.65	0.007
Chinese ethnicity English language (*n* = 82, *R* ^2^: 0.15)					
Constant	90.47		81.47	99.48	0.000
Gender	−5.87	−0.24	−11.60	−0.13	0.045
Sexual dysfunction	−9.99	−0.42	−15.49	−4.49	0.001
Indian ethnicity English language (*n* = 117, *R* ^2^: 0.11)					
Constant	84.77		82.42	87.12	0.000
Sexual dysfunction	−3.79	−0.20	−7.27	−0.30	0.033
Nerve problems	−5.70	−0.28	−9.38	−2.02	0.003
Indian ethnicity Malay language (*n* = 32, *R* ^2^: 0.45)					
Constant	113.27		98.85	127.69	0.000
Treatment	−8.26	−0.66	−11.83	−4.68	0.000
Working status	−8.53	−0.35	−15.56	−1.48	0.019

Analysis of the English-speaking group of Chinese ethnicity revealed negative association of sexual dysfunction and female gender with QOL scores (Table 5). Equation generated: QOL = 90 (constant) − 5.9 (female gender) − 10.0 (sexual dysfunction). The *R* ^2^ value was 0.15 explaining 15 % of the total QOL score. The traditional Mandarin-speaking group of Chinese ethnicity group demonstrated positive association of hyperlipidemia and negative association of glycemic control and sexual dysfunction with QOL. The equation, QOL = 71 (constant) + 4.6 (hyperlipidemia) − 1.9 (glycemic control) − 5.9 (sexual dysfunction). The *R* ^2^ value was 0.21 explaining 21 % of the total QOL score. For example, a male of Chinese ethnicity with English language as his lingua franca has HbA1c of 10 %, and complications of hyperlipidemia and sexual dysfunction will score 80 points, while a male of Chinese ethnicity with Mandarin language as his lingua franca has HbA1c of 10 %, and complications of hyperlipidemia and sexual dysfunction will score 50.7 points.

The 117 subjects from the English-speaking Indian ethnicity group show strong negative association of experiencing nerve problems and sexual dysfunction with QOL scores (Table 5). The equation formed: QOL = 85 (constant) − 3.8 (sexual dysfunction) − 5.7 (nerve problems). The *R* ^2^ value was 0.11 explaining 11 % of the total QOL score. The equation generated from analysis of the Malay-speaking Indian ethnicity group shows 113 (constant) − 8.3 (mode of treatment) − 8.5 (working status). This shows that patient on lesser type of treatment (i.e., oral vs combination of oral and insulin) and those who are working has a higher QOL score. The *R* ^2^ value was 0.45 explaining 45 % of the total QOL score.

## Discussion

The most significant new finding of the study is that the perception of QOL is different across ethnicity and the lingua franca in a population sharing the same basic socioeconomic background. This has not been demonstrated before. Analysis of this study demonstrated significant differences between the ethnic groups (Malay, Chinese and Indian) on perception of QOL. In-depth analysis of the components of QOL showed significance differences between the ethnic group’s perceptions on diet component. This demonstrates that ethnic differences do exist in a population sharing similar sociocultural contexts. This finding is similar to Singapore where a population-based study by Wee et al. in 2005 showed ethnicity as an important factor influencing QOL in people with diabetes [12]. However, a generic tool was used to measure QOL, and this could limit the sensitivity in participants with DM. Only English language tools were used in that study limiting the study population to only those proficient in English. When we compared the different ethnic groups in Malaysia, the predictors of QOL were different in the Malay ethnic group compared to the Chinese and Indian. Within the Malay ethnic group, marked differences were detected with the English-language-speaking group (ELS) versus the more traditional MLS group. The (ELS) group was primarily concerned about the presence of renal impairment, while the (MLS) group was affected by the duration of DM and sexual dysfunction. This could be that the ELS group is associated with higher education level (25 % have at least secondary education and 75 % have above secondary education) compared to the MLS group (54 % have at least secondary education and above). Higher education level is associated with better economic security and job prospects. Glasgow et al. [17] in 1997 found that lower education level and lesser income were associated with a lower QOL score. The presence of DM complications was linked to lower QOL score, especially for coexistent of microvascular and macrovascular complications [17, 20, 21]. The presence of chronic kidney disease further worsens the QOL, especially in end-stage renal failure and with initiation of dialysis [22, 23]. This is consistent with ELS group with strong negative association of renal impairment with QOL score.

The Chinese ethnic group also showed significant differences between the ELS group versus the traditional Mandarin-speaking group. The main determinant of QOL in the ELS group is sexual dysfunction versus HbA1c, hyperlipidemia and sexual dysfunction in the Mandarin-speaking group. The ELS group being more westernized in their behavior and lifestyle could have an impact on their perception compared to the more traditional Mandarin-speaking group. The presence of hyperlipidemia as a determinant of QOL scores in the Mandarin-speaking Chinese group is unique. This finding highlights the importance of the eating culture and health among the more traditional Chinese population. A population-based study in Hong Kong Chinese population found that the activity of eating was viewed as an important activity signifying good health, social bonding with family and friends [6]. This led to formation of a strong bond between the ability to eat freely, freedom to participate in such social rituals and life satisfaction affecting QOL [17, 20, 21]. The Chinese ethnic group regardless of the preferred language is severely affected by the presence of sexual dysfunction.

The predictors of QOL in the ELS Indian ethnic group were different from the Malay-speaking Indians. In the Malay-language-speaking group of Indian ethnicity, the mode of treatment and working statues explain 45 % of the total QOL score. In this group, having a permanent job is associated with better financial stability and better QOL. Poorer QOL was associated with insulin usage perhaps due to the complications of insulin. This is consistent with findings of Glasgow et al. [17] in 1996 linking insulin use to poorer QOL.

Our study highlights that perception of QOL is not only different across the ethnic groups but also among the more English-speaking and native language-speaking group within the same ethnicity. This indicates that perception of QOL is very much influenced by exposure to westernization that can be assessed by their lingua franca. Current study to look at the effect of westernization on perception of QOL among Asian population living in Australia will shed more light into this area.

In our study, we did not find any differences of QOL between subjects from private (self-paying, higher income) or government healthcare sector (free, lower income group). The mode of treatment did not affect QOL in our study group. This is consistent with the findings of the UKPDS group via two cross-sectional studies of patients in randomized controlled trials of intensive blood glucose control versus conventional control and tight blood pressure control versus less tight control, stating that the therapeutic policies had no effect on QOL [20]. Other studies done post-UKPDS also found that insulin therapy in poorly controlled type 2 DM had no adverse events on QOL [24] or even higher QOL score in the initiation of insulin phase due to relief of hyperglycemic symptoms [25]. However, there are several studies that detected a lower QOL score in subjects on insulin compared to those on oral medications [17, 26].

Complications of diabetes are associated with detrimental impact on QOL [17–20, 23]. Our study shows that among the complications, sexual dysfunction, retinopathy and nephropathy severely reduced QOL. Sexual dysfunction is also strongly negatively correlated with QOL in all the three major ethnic groups. Sexual dysfunction in this study was taken as having experienced erectile dysfunction (ED), having poor libido, premature ejaculation, vaginal dryness, dyspareunia and anorgasmia. This is consistent with findings globally that found a strong link between DM, ED and worse QOL [27, 28]. A comprehensive study done by De Berardis et al. [28] 2002, across 114 DM outpatient clinics and 112 general practitioners, found that ED affects one-third of patients with diabetes, and they have higher depressive symptoms and poorer QOL. However, this study takes into account self-reported symptoms with no clinical diagnosis, and QOL was assessed using a general assessment tool (SF-36). A year later, Person et al. in year 2003 compared impotent men with DM to those without and found more severe dysfunction and worse QOL in the group with DM [28]. Although the sample size for DM group was relatively small (*n* = 20), but a disease-specific tool was used increasing the sensitivity. In women, sexual dysfunction is frequent in patients with DM and is associated with reduction in overall QOL with 77 % having lack of libido and 38 % with vaginal dryness [29, 30]. We conclude that in a multiethnic Asian population, sexual dysfunction is highly associated with DM and in view of the detrimental effect on QOL, it is important for early detection and proper management to maintain good QOL.

However, there are limitations that need to be considered. Firstly, using a self-assessment technique by questionnaire for recruitment, only subjects who have basic education are included in the study. In view of the high literacy rate in Malaysia, our study did capture a good sample of the population [15]. Secondly, the number of Chinese-Mandarin language individuals is smaller compared to Malay/Indonesia and English group. This is mainly due to the lack of Chinese-Mandarin competent subjects in the recruitment area. Our findings are based on Malaysian population and may not be applicable to other populations in Asia.
